# Replication of the correlation between natural mood states and working memory-related prefrontal activity measured by near-infrared spectroscopy in a German sample

**DOI:** 10.3389/fnhum.2014.00037

**Published:** 2014-02-06

**Authors:** Hiroki Sato, Thomas Dresler, Florian B. Haeussinger, Andreas J. Fallgatter, Ann-Christine Ehlis

**Affiliations:** ^1^Psychophysiology and Optical Imaging, Department of Psychiatry and Psychotherapy, University of TuebingenTuebingen, Germany; ^2^Hitachi, Ltd., Central Research LaboratoryHatoyama, Japan; ^3^LEAD Graduate School, University of TuebingenTuebingen, Germany; ^4^Center of Integrative Neuroscience, Excellence Cluster, University of TuebingenTuebingen, Germany

**Keywords:** near-infrared spectroscopy (NIRS), fNIRS, prefrontal cortex, optical topography, mood, POMS, working memory, hemodynamics

## Abstract

Previous studies have suggested complex interactions of mood and cognition in the lateral prefrontal cortex (PFC). Although such interactions might be influenced by various factors such as personality and cultural background, their reproducibility and generalizability have hardly been explored. In the present study, we focused on a previously found correlation between negative mood states and PFC activity during a verbal working memory (WM) task, which had been demonstrated by using near-infrared spectroscopy (NIRS) in a Japanese sample. To confirm and extend the generalizability of this finding, we conducted a similar experiment in a German sample, i.e., participants with a different language background. Here, PFC activity during verbal and spatial WM tasks was measured by NIRS using a delayed match-to-sample paradigm after the participants' natural mood states had been evaluated by a mood questionnaire (Profiles of Mood States: POMS). We also included control tasks to consider the general effect of visual/auditory inputs and motor responses. For the verbal WM task, the POMS total mood disturbance (TMD) score was negatively correlated with baseline-corrected NIRS data mainly over the left dorsolateral PFC (i.e., higher TMD scores were associated with reduced activation), which is consistent with previous studies. Moreover, this relationship was also present when verbal WM activation was contrasted with the control task. These results suggest that the mood–cognition interaction within the PFC is reproducible in a sample with a different language background and represents a general phenomenon.

## Introduction

While we feel that our mood can influence our everyday activities to some extent, researchers have revealed a wide range of cognitive functions to be modulated by mood (Mitchell and Phillips, 2007). An example of such a mood–cognition interaction has been suggested for working memory (WM), as behavioral studies have shown that WM performance is affected by some emotional states such as withdrawal-related negative states (Gray, 2001; Shackman et al., 2006). Furthermore, the prefrontal cortex (PFC), which plays a crucial role in WM function (Kubota and Niki, 1971; Petrides et al., 1993; D'Esposito et al., 1995), has been suggested to be a brain region where cognition and emotion interact (Pessoa, 2008). In fact, while the PFC has usually been thought to be the central region underlying cognition, Nauta already suggested in 1971 that it is also related to affective and motivational responses to the person's environment based on its close association with the limbic system (Nauta, 1971; Pessoa, 2008).

Recent neuroimaging studies have investigated the impact of mood states on WM-related cortical activity. For example, a functional magnetic resonance imaging (fMRI) study showed that activity in the dorsolateral PFC (DLPFC) during a verbal (number) WM task was reduced after the participants were exposed to negative mood-inducing acute psychological stress (viewing aversive movie clips) (Qin et al., 2009). On the other hand, another fMRI study revealed that an unpleasant emotional state (induced by video clips) resulted in enhanced DLPFC activity during a verbal (letter) WM task and reduced activity during a non-verbal (face) WM task, whereas a pleasant emotional state exhibited the completely opposite pattern (Gray et al., 2002). These contradictory findings suggest a complex mood–cognition interaction in the PFC; however, an integrated theory has not been established yet. Moreover, most of the previous studies used mood-induction methods, which differ in their respective effects (Martin, 1990) and which might be different from natural mood and its effect on cognition (Parrot and Sabini, 1990). To summarize, only few aspects about the relationship between “natural moods”^1^ and WM-related PFC activity have been clarified.

To better understand the mood–cognition interaction in the PFC, we previously introduced a new approach which focused on the natural mood state in healthy participants without using a mood-induction method (Aoki et al., 2011, 2013; Sato et al., 2011). In these studies, a psychological questionnaire (Profiles of Mood States, POMS) (McNair et al., 1971; Yokoyama et al., 1990) was administered to assess the participants' natural moods during the past week, and near-infrared spectroscopy (NIRS)—a non-invasive and less body-constraint neuroimaging technique (Maki et al., 1995; Koizumi et al., 2005; Ehlis et al., 2014)—was used to measure their PFC activity during an emotionally-neutral delayed match-to-sample verbal/spatial WM paradigm (Smith et al., 1996). A correlation analysis among 29 healthy participants revealed that subjects reporting higher levels of negative mood showed lower levels of PFC activity during the verbal, but not the spatial WM task (Aoki et al., 2011). In a next step, we tried to dissociate the state-dependent effect from trait-dependent factors. Using a within-subjects design (Sato et al., 2011), experimental sessions were repeated three times at 2-week-intervals to investigate time-to-time fluctuations. The results indicated that changes in the depressed-mood score between successive sessions were negatively correlated with changes in left PFC activation for the verbal WM task (Sato et al., 2011), which is well in line with the former study (Aoki et al., 2011). Another previous study examined the contribution of personality effects (measured with the NEO Five-Factor Inventory and the Behavioral Inhibition/Activation scales) and replicated the negative correlation between negative mood scores and PFC activity for the verbal WM task even after controlling for the participants' personality traits (Aoki et al., 2013). Together, these studies strongly suggest that PFC activity during verbal WM tasks reflects the participant's natural mood-state independent of various trait factors. Furthermore, the validity of NIRS for measuring PFC activation in response to a verbal WM task was demonstrated in a simultaneous NIRS-fMRI measurement (Sato et al., 2013): A significant correlation between the NIRS signals and blood oxygenation level-dependent (BOLD) signals for the WM-related PFC activity was shown—not only for the temporal changes, but also for the amplitude of the signal response (Sato et al., 2013).

In the present study, we aimed to replicate our previous findings on a mood–cognition interaction in the PFC for a verbal WM task (Aoki et al., 2011) in a German sample, i.e., participants with a different language background while also striving to improve previous methodical weaknesses: First, we find it necessary to confirm the reproducibility of the findings when the PFC activity is derived from a contrast with a suitable control condition in an improved task design. Because the previous studies only used activation values relative to baseline, possible confounds (input of visual stimuli, reaction by button press, etc.) could not be ruled out. Second, it is important to determine whether the phenomenon can be replicated in other samples with a different cultural and ethnical background. In particular, because a verbal function is the object of our study, the replication in a sample with a different language background is necessary to show the robustness of this phenomenon. Indeed, the Japanese letters used in our previous studies represent a minority in the world; besides, it is still difficult to determine the genetic affiliation of Japanese language to other languages or language families (Teruya, 2004). In this study, we conducted an experiment in a German sample whose language belongs to the major language family of “Indo-European languages” using Latin alphabet letters. Although we usually assume that there are no significant cultural/language-related differences in cognitive neuroimaging data, it is worth replicating the results in a sample with a different language background to make our knowledge more practical.

## Materials and methods

### Participants

Thirty-one healthy German volunteers participated in this study (16 females and 15 males, mean age ± SD = 28.0 ± 7.1 years). All of them reported no history of neurological or psychiatric disease and gave their written informed consent to participate in this study, which was approved by the Ethics Committee of the University Hospital Tuebingen. All procedures were in line with the Declaration of Helsinki in its latest version. Both males and females were included in this study because our previous study did not reveal any gender differences for the main result of a correlation between negative mood and PFC activity (Aoki et al., 2011). After we excluded data from four participants with increased depression scores (BDI scores > 14) and one participant with poor task performance (i.e., accuracy <60%), data from 26 participants (13 females and 13 males, all right-handed except for one male) with a mean age of 25.9 ± 4.6 years (age range: 20–42) were further analyzed. All of them had a higher education (of at least 13 years; “Abitur” in German).

### Working memory tasks

**Figure 1:**
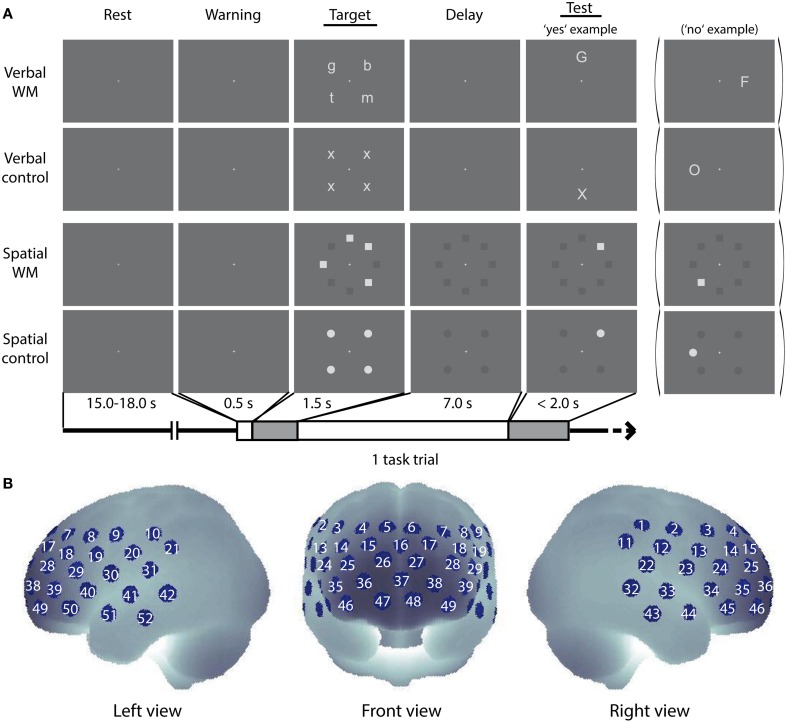
**Cognitive tasks and NIRS measurement positions. (A)** Schematic diagram of a task trial sequence. Example images for the verbal WM task, the control task for verbal WM, the spatial WM task, and the control tasks for spatial WM are shown. **(B)** Arrangement of the NIRS measurement channels (52 measurement positions) in the Montreal Neurological Institute (MNI) space (see Materials and Methods, for more detail).

WM refers to the temporary retention of information to perform cognitive activities (Baddeley, 1983; D'Esposito, 2007). Here, we used verbal and spatial delayed match-to-sample WM tasks (Smith et al., 1996) as previously applied (Aoki et al., 2011, 2013; Sato et al., 2011) (Figure 1A).

In the verbal WM task, four lowercase letters were presented at four peripheral locations as a target stimulus (Target), and a capital letter was presented at one of the other four peripheral locations as a test stimulus (Test) (see Target and Test stimuli for verbal WM condition in Figure 1A). We pseudo-randomly selected and arranged four sets from eight letters (b, f, g, h, m, n, p, t) for the Target, not to make up any meaningful words. The participants were asked to judge whether the character presented as Test corresponded to any of the Target characters and to respond as quickly as possible by pressing a button (“yes”—right index finger, “no”—right middle finger). Since the characters in Target and Test were presented in different letter-types (lowercase/capital), participants had to decide by using the phonetic information (i.e., the phonological loop). In the verbal control (v-control) task, four “x”s were presented at the four fixed locations as Target, and either an “X” or “O” was presented at one of the other four locations as Test. The participant had to decide whether the Test character was “X” (Yes) or “O” (No). This v-control task was designed to match the verbal WM task in visual/auditory inputs and motor response with less WM load.

In the spatial WM task, the Target was given by the locations of four squares irregularly arranged at eight peripheral locations. The arrangement of squares was organized in a complex way (e.g., no more than three successive squares, no meaningful arrangement like large square or rhombus). After an arrangement was presented as Test, the participant was asked to judge whether the Test square location was identical to any of the Target square locations. In the spatial control (s-control) task, four circles arranged to make a regular square were presented as Target, and a circle was presented at a location among the eight peripheral locations as Test. The participant had to decide whether the location of the Test circle was identical to any of the Target circle locations. Responses were indicated as in the verbal task.

The software *Presentation* (Neurobehavioral Systems, Inc., U.S.A.) was used for stimuli presentation and reaction time (RT) recording. Each WM task trial was as follows (see Figure 1): First, a central fixation cross (30% black) turned white 500 ms before the Target onset with an auditory cue (1000 Hz sine wave of 100 ms duration) as a warning signal. Then the Target was presented for 1500 ms, followed by a delay period of 7000 ms with the white fixation cross. During the delay period for the spatial WM task, positions of the eight peripheral squares were also shown in dark-gray (75% black). In the same way, positions of the four peripheral circles were shown for the delay period in the s-control task. Test, indicated by an 800 Hz sine wave of 100 ms duration, was presented until the participant responded (maximum: 2000 ms). All stimuli in Target and Test were shown in light-gray (20% black) at a location among the eight peripheral locations, and the inter-trial-intervals with the central fixation cross (30% black) were randomly varied between 15 and 18 s. A dark-gray background (70% black) was shown throughout.

Participants performed the verbal and spatial WM tasks in different runs. In both verbal WM and spatial WM runs, 8 WM task trials and 8 control trials were presented in a pseudo-randomized order (16 task trials in total for each run). In addition, “yes” and “no” trials were pseudo-randomized to be the same number (i.e., 4 trials for “yes” and the other 4 for “no” in each condition). The order of runs (verbal WM run and spatial WM run) was counterbalanced across participants.

### Procedure

Before the NIRS measurement, the participants' natural mood was assessed by using a short form of the POMS (McNair et al., 1971) in its German version (Bullinger et al., 1990). The participants rated 35 mood-related adjectives on a 7-point scale ranging from 0 (“not at all”) to 6 (“extremely”) based on how they had been feeling in the past week including the measurement day. While the German version of the POMS consists of four identifiable mood states (depression/anxiety, vigor, fatigue, and discontent), we defined the total mood disturbance (TMD) score as a general negative mood score calculated as the sum of the negative mood scores (depression/anxiety, fatigue, and discontent) minus the positive mood score (vigor). As previous studies showed that PFC activity correlated negatively with negative moods while tending to correlate positively with positive moods (Aoki et al., 2011, 2013), TMD would be an appropriate mood index which is expected to correlate negatively with PFC activity.

Thereafter, PFC activity during the performance of the WM tasks was measured by using a multi-channel NIRS (optical topography) system (ETG-4000, Hitachi Medical Corporation, Japan). The system uses continuous wave laser diodes with two wavelengths (695 and 830 nm) as light sources. Optical fibers are used both for irradiation and for detection of near-infrared light. Seventeen irradiation positions and 16 detection positions were arrayed in a 3 × 11 lattice pattern with 30 mm separation, forming 52 measurement channels (Figure 1B). The average power of each wavelength light was 1.5 mW at the irradiation point, which was modulated at different frequencies to be separated for wavelengths and sources in the detection process. The reflected light at a position 30 mm apart from the irradiation position was detected by avalanche photodiodes and recorded in the computer with a sampling rate of 10 Hz.

**Figure 2:**
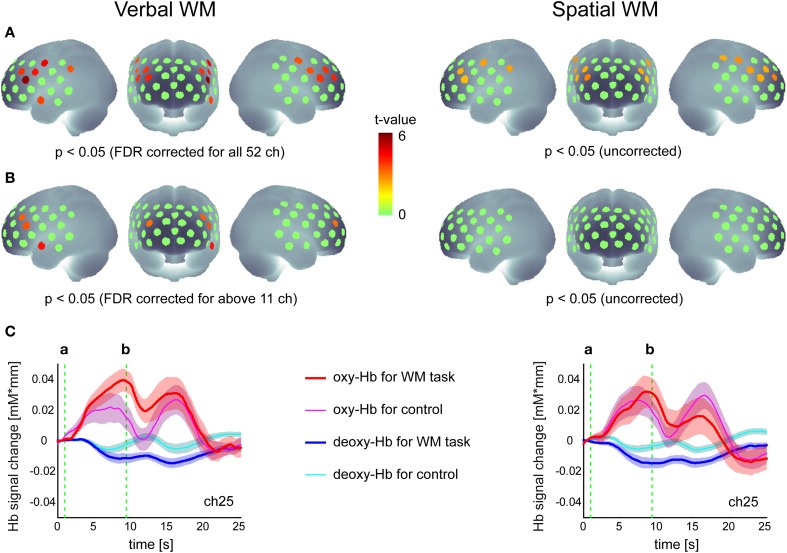
**Hemodynamic responses to the verbal and spatial WM tasks. (A)** Activation t-maps of oxy-Hb signal increase during WM tasks compared to the baseline (zero level). **(B)** Activation t-maps of oxy-Hb signal increase during WM tasks compared to the signal during the control task. In both maps, the Student's *t*-value is indicated by a color scale for channels which meet the *p*-value criteria described below each map. **(C)** Time courses of Hb-signal in a representative channel (Ch 25). These time courses represent the grand average across all participants (*N* = 26) with standard error (transparent color region). The green vertical lines show the time of (a) Target onset and (b) Test onset, respectively.

**Figure 3:**
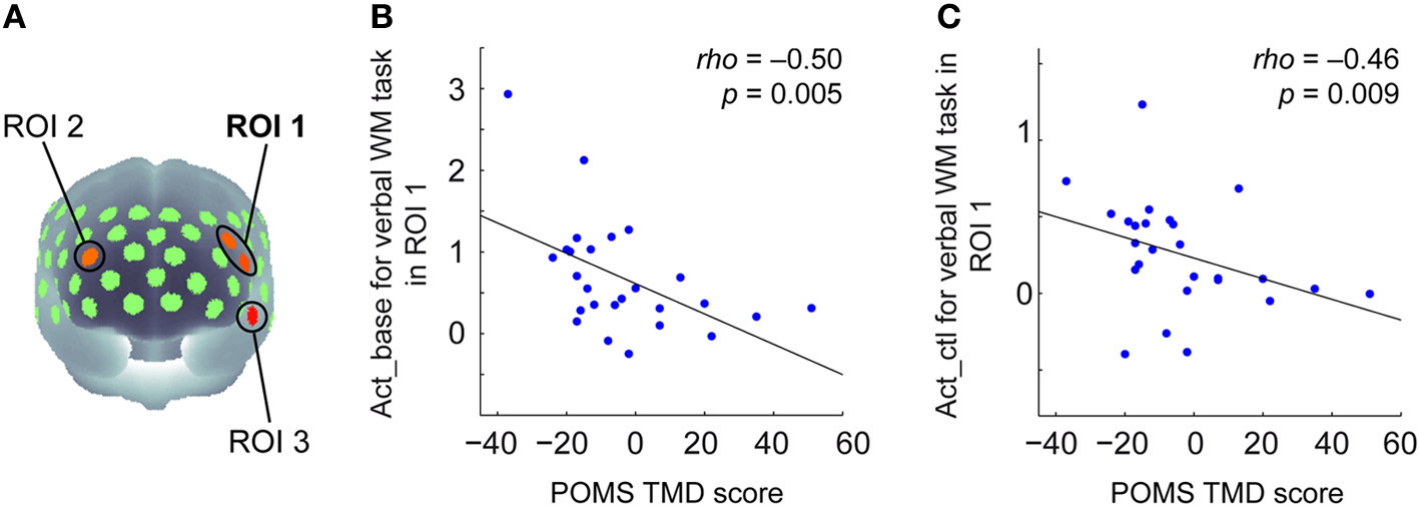
**Correlation between POMS TMD scores and activation values in oxy-Hb signals for the verbal WM task. (A)** Three ROIs determined by the activation t-maps for the verbal WM task compared to the v_control task (Figure 2B). **(B)** Scatter plot showing a significant negative correlation between the POMS TMD score and the Act_base in ROI-1. **(C)** Scatter plot showing a significant negative correlation between the POMS TMD score and the Act_ctl in ROI-1.

To obtain the anatomical position of the NIRS channels, we measured the three-dimensional coordinates of the optode positions in four German volunteers by using a neuro-navigation system (LOCALITE GmbH, St. Augustin, Germany). The obtained optode coordinates were transferred from the volunteers' native MRI space to the standard Montreal Neurological Institute (MNI) space by applying normalization procedure of Statistic Parametric Mapping 8 (SPM8, http://www.fil.ion.ucl.ac.uk/spm/software/spm8/). The normalized coordinates were used in the probabilistic registration method (Okamoto et al., 2004; Okamoto and Dan, 2005) to estimate the brain region for each measurement channel and to generate 3-D topographical maps (Figures 1–3). The averaged MNI coordinates on the brain are listed with the corresponding Brodmann Area (BA) number (Rorden and Brett, 2000) and the anatomical label determined by Automated Anatomical Labeling (AAL) (Tzourio-Mazoyer et al., 2002) in the Supplementary Material (Table S1).

### Data analysis

For NIRS data analysis, we used MATLAB (The MathWorks, Inc., U.S.A.) and the plug-in-based analysis software Platform for Optical Topography Analysis Tools (developed by Hitachi, CRL).

For each NIRS channel, the optical data for the two wavelengths were transformed into a time series of hemoglobin (Hb) signals (oxy-Hb and deoxy-Hb signals) on the basis of the modified Beer–Lambert law (Delpy et al., 1988; Maki et al., 1995). These signals were expressed as the product of the changes in hemoglobin concentration (mM) and optical path length (mm) in the activation region (effective optical path length). A butterworth band-pass filter (0.013–0.80 Hz) was then applied to the Hb signals to remove low-frequency drift/oscillation and high-frequency system noise. The time-continuous data of Hb signals were divided into 25.5-s task blocks, which consisted of a 1-s pre-task period (starting 1 s before Target onset), an 8.5-s task period (during the 1.5-s Target presentation and the 7-s delay period) and a 16-s post-task period (starting after Test onset). Each task block was baseline-corrected by subtracting the average value of the pre-task period. Here, we especially focused on the oxy-Hb signal in the analysis as previous studies underlying the present work found their main effects in this chromophore (Aoki et al., 2011, 2013; Sato et al., 2011). Note that we did not include data from error-response trials for the further analysis.

To evaluate WM-associated PFC activity, we calculated two kinds of activation values for each channel and participant. The first activation value (Act_base) contrasts the mean oxy-Hb signal values during a 6-s “activation period” (starting 5 s after Target onset) with the baseline. The activation period was determined to investigate the signal change induced by the encoding and maintenance processes in WM function, avoiding confounding effects related to the motor response and proactive interference (D'Esposito et al., 2000). These oxy-Hb signal values were then averaged across correct-response trials and divided by their standard deviation across trials. The second activation value (Act_ctl) contrasts WM activation with the control task: The differences between the mean values for a WM task minus those for the control task were averaged across correct-response trials and divided by the standard deviations across trials. These activation values, which take the within-participant reproducibility into consideration, should be more reliable than a mean value from a simply-averaged waveform which is easily influenced by an accidental large response-like change in a single trial.

In the subsequent group analysis, we performed channel-wise statistical tests on the activation values to determine the activation regions. First, significant PFC activation relative to the baseline was assessed by using one-sample *t*-tests (one-tailed) of Act_base in all 52 channels. Next, significant PFC activation relative to the control task was assessed in a similar way using Act_ctl for the activated channels determined by the former analysis in Act_base. In these channel-wise analyses, we used the false discovery rate (FDR) method to correct for multiple comparisons among channels (Benjamini and Hochberg, 1995; Singh and Dan, 2006). Based on the activation map for Act_ctl, we selected significantly-activated channels as regions of interests (ROIs). In each ROI, correlation coefficients between mood states (TMD scores) and activation values were calculated using Spearman rank correlation coefficients (*rho*) as the association may not be necessarily linear. Here, we tested significance of the negative correlation (one-tailed) with the FDR correction for multiple comparisons for the number of ROIs. To further examine whether the correlations were influenced by confounding factors such as age, gender, and task performance (accuracy and reaction time), we performed partial correlation analyses. In addition, correlation coefficients between behavioral data (accuracy and reaction time) and the TMD score were also examined to consider mood effects on the behavioral level.

## Results

### Behavioral data

The accuracy and RT data are shown in Table 1. Two-Way repeated-measures ANOVAs were used to examine the effects of WM load (WM task/control task) and content (verbal/spatial). For accuracy, a main effect of WM load was found [*F*_(1, 25)_ = 11.82, *p* < 0.01], which indicates that the control tasks were easier to solve, as expected. In contrast to that, no significant results were obtained for content [*F*_(1, 25)_ = 0.41, *p* = 0.53] or the interaction of WM load × content [*F*_(1, 25)_ = 0.37, *p* = 0.55]. For RT, a main effect of WM load was found [*F*_(1, 25)_ = 49.11, *p* < 0.001] without a main effect of content [*F*_(1, 25)_ = 0.38, *p* = 0.54], which indicates that both WM tasks were associated with longer reaction times than the control tasks. A significant interaction was also found [*F*_(1, 25)_ = 10.53, *p* < 0.01], indicating that the RT difference between the WM and control tasks was larger for the verbal WM condition (mean difference in the RTs: 161 ms) compared to that for the spatial WM condition (mean difference in the RTs: 65 ms).

**Table 1:** **Descriptive statistics of task performance (*N* = 26)**.

		**Accuracy (%)**		**Reaction time (ms)**	
		**Mean**	***SD***	**Mean**	***SD***
Verbal WM	Task	94.2	8.1	799	172
	control	98.6	4.1	638	100
Spatial WM	Task	92.3	12.3	740	137
	control	97.9	4.1	675	135

The correlation coefficients between the accuracies and TMD scores were not significant (verbal WM: *rho* = 0.09, *p* = 0.65; spatial WM: *rho* = 0.25, *p* = 0.22). The correlation coefficients between the RTs and TMD scores were also not significant, but displayed a tendency toward a negative association (verbal WM task: *rho* = −0.33, *p* = 0.10; spatial WM task: *rho* = −0.34, *p* = 0.09), i.e., participants who had higher TMD scores showed lower RTs (faster responses) for both WM tasks.

### PFC activity during WM tasks

Activation maps in the group analysis are shown in Figure 2. For the verbal WM task, the increase in oxy-Hb signal relative to baseline was significant in 11 channels in the bilateral PFC (*p* < 0.05, FDR corrected for 52 channels). Four of these channels also showed significant activation in contrast to the v-control task (*p* < 0.05, FDR corrected for 12 channels). The representative time courses (ch25) shown below the activation map (Figure 2) indicate clear responses in the oxy-Hb signal after the presentation of Target stimuli, where the responses for the WM task were larger than those for the control task.

This activation pattern was not evident for the spatial WM task, where no significant increases in oxy-Hb signal relative to the baseline were found when the threshold was corrected for the number of channels. Although the uncorrected activation maps indicated oxy-Hb signal increases in the PFC region relative to the baseline (similar to the result for the verbal WM task), no significant differences were found when contrasted with the s-control task (Figure 2). While the time courses (ch25) showed a similar response to the Target stimuli as shown in the verbal WM condition, the standard error (inter-participant variation) was larger and there was no clear difference from the response to the control task. As activation did not differ between WM and control task, we did not include the spatial WM data in the subsequent correlation analysis.

### Correlation of mood states with PFC activity for the verbal WM task

TMD scores measured by POMS ranged from −37 to 51 across participants (Mean = −3.65, *SD* = 19.36). To examine the relationship between the negative mood state and PFC activity for the verbal WM task, the correlation coefficients between TMD scores and activation values were calculated for the ROIs. Here, we defined the WM-related activation channels determined by contrasting to the control task (Figure 2B) as the ROIs (Figure 3A), and the mean activation values of the two channels (channels 18 and 29) were used in the correlation analysis for ROI-1.

The results of the correlation analysis are summarized in Table 2. In ROI-1 in the left PFC, which consists of channels 18 (“Frontal_Mid_L,” BA45) and 29 (“Frontal_Inf_Tri_L,” BA45), both Act_base (activation value relative to baseline) and Act_ctl (activation value relative to the v-control task) showed significant negative correlations with TMD scores (*p* < 0.05, FDR corrected for 3 ROIs) (Table 2 and Figures 3B,C). These correlations remained significant even when the control variables (gender, age, accuracy and RT for the verbal WM task) were entered into a partial correlation analysis (Act_base: *rho* = −0.50, *p* = < 0.01; Act_ctl: *rho* = −0.42, *p* < 0.05). Further, when analyzed separately by gender, females and males showed equivalent correlation coefficients for Act_ctl (female: *rho* = −0.45, *p* = 0.12; male: *rho* = −0.43, *p* = 0.15) although they did not reach statistical significance due to small sample sizes. On the other hand, ROI-2 in the right PFC (channel 25, “Frontal_Mid_L,” BA46) also showed a significant negative correlation for Act_base (*p* < 0.05, FDR corrected for 3 ROIs) but not for Act_ctl (*p* > 0.05, FDR corrected for 3 ROIs). In addition, ROI-3 in the left temporal pole (channel 51, “Temporal_Pole_Sup_L,” BA38) did not show any significant correlation for either Act_base or Act_ctl (*p* > 0.05, FDR corrected for 3 ROIs).

**Table 2:** **Correlation coefficient (*rho*) between TMD scores and activation values in ROI**.

**ROI (channel No.)**	**Anatomical info**.	**Act_base**		**Act_ctl**	
		***rho***	***p***	***rho***	***p***
ROI-1 (18&29)	“Frontal_Mid_L”	**−0.50**	**0.005**	**−0.46**	**0.009**
	“Frontal_Inf_Tri_L”				
ROI-2 (25)	“Frontal_Mid_R”	**−0.43**	**0.014**	−0.35	0.041
ROI-3 (51)	“Temporal_Pole_ Sup_L”	−0.06	0.384	−0.33	0.048

Uncorrected p-values are shown for the correlation coefficients. Significant rho- and p-values (p < 0.05 with FDR correction) are indicated by bold letters.

As the waveform of oxy-Hb signals showed a bi-phasic pattern (Figure 2C), we additionally conducted the same correlation analysis using different activation values for the second peak (using activation period: 11–17 s after Target onset) (Table S2). As a result, the activation value relative to the baseline (Act2_base) in the left PFC (ROI-1) still showed a significant negative correlation (*p* < 0.01, FDR corrected for 3 ROIs) while the activation value relative to the control task (Act2_ctl) did not reach statistical significance (*p* > 0.05, FDR corrected for 3 ROIs). Activation values in ROI-2 and ROI-3 showed the same tendency of negative correlations with no statistical significance.

## Discussion

In the present NIRS study, we aimed to confirm and extend the generalizability of findings about an association between negative mood states and PFC activity for a verbal WM task as demonstrated in Japanese samples (Aoki et al., 2011, 2013). We could show that negative mood (TMD score evaluated by POMS) was negatively correlated with left-hemisphere PFC activity during a verbal WM task in a German sample. This was also evident when subtracting activation in the v-control task from the WM task. That is, participants with higher TMD scores showed less PFC activity during the verbal WM task, which is consistent with the above-mentioned findings. To our knowledge, this is the first study that replicates this relationship in a sample with a different language background, suggesting that the mood–cognition interaction in the PFC represents a general phenomenon that is reproducible across samples with different cultural and ethnical background.

### PFC activity during WM tasks

The oxy-Hb signals increased during the verbal WM task in the bilateral PFC (Figure 2). The activated cortical region and the shape of Hb-signal changes do not only resemble those in the published studies (e.g., Aoki et al., 2011), but also those in corresponding fMRI studies (D'Esposito, 2007). The contrast with the control task showed more localized activation around the bilateral middle frontal gyrus (BA46) and the triangular part of left inferior frontal gyrus (BA45) consistent with available PET/fMRI studies (Petrides et al., 1993; D'Esposito et al., 1995; Swartz et al., 1995; Smith et al., 1996; Smith and Jonides, 1999; Walter et al., 2003). This result suggests that the control task was well-chosen to capture the WM relevant PFC regions. Although some studies suggested that extracranial hemodymanic changes such as skin blood flow considerably influence the forehead NIRS signals (Takahashi et al., 2011; Kirilina et al., 2012), a simultaneous NIRS-fMRI study demonstrated that in a comparable WM task Hb-signals were significantly correlated with gray matter BOLD signal rather than with soft tissue or skin blood flow signals (Sato et al., 2013).

In contrast to the verbal WM task, the increase in the oxy-Hb signal for the spatial WM task was not significant in a conservative analysis with FDR correction, although we observed a similar activation region when compared to the baseline without multi-channel correction (Figure 2). This result was not consistent with our previous NIRS studies which showed the same activation for the spatial as in the verbal WM task (Aoki et al., 2011). As the behavioral accuracy and RT data did not reveal differences between tasks, a difference in difficulty cannot account for this discrepancy. However, the RT data indicates that the difference between WM and control task was less pronounced for the spatial WM task, which could explain the absence of a significant finding for spatial WM. In addition, as the four circle positions in the Target image were fixed for the s-control task to minimize WM load, it may have been seen as a totally different task by the participant. That is, both the WM task and the control task could activate regions involved in “set-shifting” (Konishi et al., 1999).

### Correlation of mood states with PFC activity for the verbal WM task

We found a significant negative correlation of the TMD score with both Act_base (activation relative to the baseline) and Act_ctl (activation relative to the v_control task) for the verbal WM task in the left PFC (around BA45), as found in the aforementioned NIRS studies, demonstrating the reproducibility of the relationship between negative mood and WM-dependent PFC activity. This result extends our knowledge for the following reasons: First, though to a lesser extent, it was also found for Act_ctl, which suggests that the main component of the correlated NIRS signal indeed stems from a verbal WM function rather than general task demands (e.g., visual/auditory inputs and execution of the motor response). Second, the significant correlation was demonstrated in a localized activation area within the left PFC, which overlaps with the main verbal WM-related regions identified by other modalities (Petrides et al., 1993; Smith et al., 1996; Walter et al., 2003). This can be interpreted as evidence against a critical distortion of our findings by confounding skin blood flow changes which are supposed to appear across large parts of the measurement array (Kohno et al., 2007). According to the “prefrontal asymmetry” hypothesis (Davidson, 1992), positive (approach-related) and negative (withdrawal-related) affect are predominantly associated with the left (verbal function) and right (spatial) hemisphere, respectively (Gray, 2001; Gray et al., 2002). Based on this idea, our results (i.e., decreased left PFC activity) might reflect a reduced positive mood state rather than increases in negative mood. Third, we could confirm the replication of our previous findings in a sample with a different language background. This suggests that the relationship between mood and verbal WM function is general regardless of language. Previous studies have shown cultural influences on the brain activation related to some social cognitive tasks (Chiao et al., 2010; Chiao and Immordino-Yang, 2013). However, up to now, no studies have shown such cultural influences on the lateral PFC during emotionally-neutral cognitive tasks such as our WM tasks, as far as we know. This might indicate that PFC activity for emotionally-neutral cognitive tasks is independent of cultural backgrounds. Although we could not directly compare the activation pattern between Japanese and German samples, cultural differences in PFC activity during emotionally-neutral cognitive tasks might be an important topic to determine the range of cultural effects on the brain. In addition, it should be emphasized that we could also replicate the significant correlation using partial correlation analysis with control variables of gender, age, and task performance. This indicates that the mood–cognition relationship in the PFC cannot be explained by any of the control variables.

Our results are basically in line with an fMRI study by Qin et al. (2009) who demonstrated that experimentally-induced acute stress was accompanied by a decrease in verbal WM-related DLPFC activity. In addition, both studies did not reveal correlational patterns with behavioral data, possibly due to ceiling effects of the task performance. This means that it is not clear whether the negative mood states hinder the PFC function or improve its efficiency. To increase the number of task trials or to make the task more difficult would be a possible solution to reveal the relationship between behavioral measures and mood states. Actually, the previous study showed that participants with the strongest stress response slowed down the most (supporting a hindrance of the PFC) (Qin et al., 2009), while our results indicated a tendency for participants with higher negative mood scores to show faster responses (supporting an improvement of PFC function). Nevertheless, these results suggest that decreased PFC activity can be a neural marker of heightened stress or increased negative mood states and seems to be more sensitive than behavioral performance.

### Limitation and future perspectives

The first limitation of the present study concerns a failure to detect PFC activation for the spatial WM task using a corrected significance threshold. As the previous NIRS study showed a significant correlation between mood and PFC activity only for the verbal WM task but not for the spatial WM task while the two tasks induced almost equivalent PFC activities (Aoki et al., 2011), it would be worth investigating the difference of the two tasks in order to further identify the cognitive factors underlying the mood–cognition interactions.

Next, we could not directly compare the basic NIRS results between German and Japanese samples, because some differences in methods and theoretical considerations did not allow such a procedure. First, differences in the applied paradigms such as the control task and the repetition time restrict comparability and would make it difficult to attribute any potential group differences to a specific factor. Additionally, results from different NIRS machines should not be directly compared as general equivalence in technical parameters, etc. of the machines had not been tested. Second, a direct comparison was not intended as no hypotheses exist why and how these samples differ. Indeed, the main purpose of this study was to replicate the relationship between PFC activity and mood scores, which was successfully done regardless of some cultural/ethnical differences and task modifications. Only testing the samples using exactly the same paradigm parameters and the same NIRS machine would help to systematically explore potential differences between both samples.

Finally, the present study aimed at replicating previous NIRS findings and was not specifically designed to further explain the cognitive or physiological mechanisms underlying the observed mood–PFC relationship. Indeed, we found a similar correlation pattern for the second peak of PFC activation corresponding to the retrieval phase (Table S2), while focussing on the activity for encoding and maintenance phases. This might suggest the necessity to resolve the mood-related WM function into cognitive sub-processes in the future. Furthermore, it is necessary to investigate the effects of some other physiological parameters on the mood–PFC correlation to advance our knowledge about the causal relationship between natural mood and PFC activity.

## Conclusion

The present study aimed at replicating a previous finding on an interaction between mood states and PFC activity for a verbal WM task, which was found by a NIRS study in a Japanese sample. Conducting a comparable experiment in a German sample, we could indeed confirm this finding; negative mood scores were negatively correlated with verbal WM-related PFC activity across participants, even when the activation values were contrasted with an adequate control task. Although we failed to observe reliable PFC activity for the spatial WM task, it is important to note that the relationship between negative mood and PFC activity for the verbal WM task could be confirmed in a sample with a different language background. Our results suggest that the mood–cognition interaction within the PFC is reproducible and represents a general phenomenon.

### Conflict of interest statement

The authors declare that the research was conducted in the absence of any commercial or financial relationships that could be construed as a potential conflict of interest.

## Footnotes

^1^“Natural moods” are defined as relatively low-intensity and diffuse affective states without a striking preceding cause (Forgas, 1995), being different from emotion and experimentally induced moods.

## Supplementary material

Table S1 **Estimated location of each NIRS channel on normalized brain image. Mean MNI coordinates across participants are shown with the corresponding Brodmann area (BA) number (Rorden and Brett, 2000) and the anatomical label determined by Automated Anatomical Labeling (AAL) (Tzourio-Mazoyer et al., 2002)**.

Table S2 **Correlation coefficient (rho) between TMD scores and activation values (Act2_base: contrasted to baseline; Act2_ctl: contrasted to the control task) for the second peak (activation period: 12–18 s after the Target onset)**.
