# Supplementary material for: Replication of the correlation between natural mood states and working memory-related prefrontal activity measured by near-infrared spectroscopy in a German sample
Source: Front Hum Neurosci. 2014 Feb 6;8:37. doi: 10.3389/fnhum.2014.00037 (PMC3915104; doi:10.3389/fnhum.2014.00037)
Supplement: Table S2 — Correlation coefficient (rho) between TMD scores and activation values (Act2_base: contrasted to baseline; Act2_ctl: contrasted to the control task) for the second peak (activation period: 12–18 s after the Target onset). [file DataSheet2.PDF]

Table S2 Correlation coefficient (*rho*) between TMD scores and activation values (Act2_base: contrasted to baseline; Act2_ctl: contrasted to the control task) for the second peak (activation period: 12-18 s after the Target onset)

ROI. (channel No.)	Act2_base		Act2_ctl	
	rho	*p*	*rho*	*p*
ROI-1 (18&29)	−0.57	**0.001**	−0.34	0.043
ROI-2 (25)	−0.38	0.027	−0.37	0.030
ROI-3 (51)	−0.20	0.169	−0.37	0.032

Uncorrected *p*-values are shown for the correlation coefficients. Significant *rho* and *p* values ($p < 0.05$ with FDR correction) are indicated by bold letters.
